# Supplementary material for: Significance of P53-Binding Protein 1 as a Novel Molecular Histological Marker for Hypopharyngeal Squamous Neoplasms
Source: Cancers (Basel). 2024 Aug 28;16(17):2987. doi: 10.3390/cancers16172987 (PMC11394016; doi:10.3390/cancers16172987)
Supplement: Supplementary file 1 [file cancers-16-02987-s001.zip › cancers-3145210-supplementary.pdf]

Table S1

The number of sites, cases and types of p53-binding protein 1 (53BP1) and Ki67 expression in hypopharyngeal lesions in this study.

Lesions<br><br>sites (cases)	Counted<br>nuclei	Stable 53BP1		Unstable 53BP1		Ki67<br>Positive nuclei	53BP1/Ki67<br>colocalization
		n=0	n=1,2	n>3	LF		
Non tumor<br><br>38 (38)	11646	11011<br>(94.24%)<br>(38)	348<br>(3.04%)<br>(38)	828<br>(6.92%)<br>(15)	45<br>(0.34%)<br>(23)	828<br>(6.92%)<br>(35)	45<br>(0.34%)<br>(16)
LD<br><br>14 (14)	6073	5343<br>(86.73%)<br>(14)	331<br>(5.87%)<br>(14)	576<br>(9.77%)<br>(10)	63<br>(1.13%)<br>(9)	576<br>(9.77%)<br>(14)	63<br>(1.13%)<br>(10)
HD<br><br>8 (8)	3376	3099<br>(81.61%)<br>(8)	369<br>(10.04%)<br>(8)	314<br>(8.58%)<br>(7)	59<br>(1.61%)<br>(8)	314<br>(8.58%)<br>(8)	59<br>(1.61%)<br>(7)
SCC<br>Surface<br>30 (30)	9727	7914<br>(83.44%)<br>(30)	838<br>(8.23%)<br>(30)	912<br>(9.61%)<br>(27)	232<br>(1.32%)<br>(27)	912<br>(9.61%)<br>(30)	232<br>(1.32%)<br>(24)
SCC<br>invasive front<br>35 (35)	15472	12825<br>(76.81%)<br>(35)	1215<br>(10.35%)<br>(35)	2055<br>(12.75%)<br>(28)	407<br>(3.26%)<br>(29)	2055<br>(12.75%)<br>(35)	407<br>(3.26%)<br>(30)
				p<0.0001			p<0.0001

LD: low-grade dysplasia; HD: High-grade dysplasia; SCC: Squamous cell carcinoma. 53BP1/Ki67: 53BP1 and Ki67.
